# Harnessing Nanoplasmonics: Design Optimization for Enhanced Optoelectronic Performance in Nanocrystalline Silicon Devices

**DOI:** 10.3390/mi16050540

**Published:** 2025-04-30

**Authors:** Mohsen Mahmoudysepehr, Siva Sivoththaman

**Affiliations:** 1 Power Solutions Group, Onsemi, Scottsdale, AZ 85250, USA; 2 Department of Electrical Engineering, University of Waterloo, Waterloo, ON N2L 3G1, Canada

**Keywords:** nanoplasmonics, metallic nanoparticle arrays, nanocrystalline silicon thin films, light trapping, finite-difference time-domain (FDTD) simulation, surface plasmon resonance, plasmon hybridization, waveguide modes, optoelectronic devices

## Abstract

Nanoplasmonic structures have emerged as a promising approach to address light trapping limitations in thin-film optoelectronic devices. This study investigates the integration of metallic nanoparticle arrays onto nanocrystalline silicon (nc-Si:H) thin films to enhance optical absorption through plasmonic effects. Using finite-difference time-domain (FDTD) simulations, we systematically optimize key design parameters, including nanoparticle geometry, spacing, metal type (Ag and Al), dielectric spacer material, and absorber layer thickness. The results show that localized surface plasmon resonances (LSPRs) significantly amplify near-field intensities, improve forward scattering, and facilitate coupling into waveguide modes within the active layer. These effects lead to a measurable increase in integrated quantum efficiency, with absorption improvements reaching up to 30% compared to bare nc-Si:H films. The findings establish a reliable design framework for engineering nanoplasmonic architectures that can be applied to enhance performance in photovoltaic devices, photodetectors, and other optoelectronic systems.

## 1. Introduction

The rapid evolution in design concepts for micro- and nanoscale semiconductor devices has significantly broadened their scope of their application in photovoltaic energy conversion, photodetection, and sensing technologies. Among these advancements, nanoplasmonic architectures offer a unique approach to surpass the intrinsic limitations associated with light trapping, especially critical for ultrathin semiconductor films [1,2,3,4,5]. Early work in the field, dating back to the 1990s, demonstrated that even ultrathin metallic layers could induce localized surface plasmon resonances (LSPRs) that enhance photovoltaic performance by increasing light scattering, near-field intensities, and waveguide mode coupling [6,7,8,9,10,11].

Metallic nanoparticles integrated onto active absorber layers, such as nanocrystalline silicon (nc-Si:H), demonstrate remarkable potential by facilitating intense local electromagnetic fields, increasing forward scattering, and effectively coupling incident photons into waveguide modes. Early research identified that localized surface plasmon resonances, induced by optimized metallic arrays, substantially improve optical absorption and efficiency, thus enhancing device performance across the broadband spectrum.

Expanding beyond photovoltaics, these nanoplasmonic structures serve as versatile platforms for multifunctional optoelectronic devices, enabling enhanced broadband response and heightened spectral sensitivity [12,13,14]. For instance, tailored nanoparticle arrays have been successfully deployed in photodetectors, significantly boosting spectral responsivity through optimized plasmon-induced scattering and near-field enhancement [15,16]. Similarly, in light-emitting devices and integrated photonic circuits, precise control over plasmonic resonance frequencies and electromagnetic field distributions provides pathways to enhanced functionalities and heightened device efficiency [17,18]. By engineering the nanoparticle geometry, spacing, and surrounding dielectric environment, one can manipulate the local density of optical states and achieve controlled emission enhancements—an approach that holds promise for developing next-generation LEDs and integrated photonic circuits [19]. Additionally, these architectures are ideally suited for sensor applications, where surface-enhanced Raman scattering (SERS) and refractive index-based sensing can be exploited to yield for high-sensitivity detection.

In this work, we utilized finite-difference time-domain (FDTD) simulations to systematically parameterize the design of metallic nanoparticle arrays by integrating them into hydrogenated nanocrystalline silicon (nc-Si:H) thin films and studying the optical behavior. By rigorously optimizing key parameters, including nanoparticle type (Ag, Al), nanoparticle geometry, inter-particle spacing, spacer material, and absorber layer thickness, pathways for enhanced optical absorption are identified and a design framework is established for nc-Si:H-based photovoltaic, detection, sensing, and emission devices.

## 2. Materials and Methods

The choice of nanoplasmonic material is an important consideration [3,4,5,6,20,21]. While Ag and Au are known for their strong resonances in the visible range [22,23], alternative materials like Al and Cu can also offer cost or spectral advantages under certain conditions. Next, parametric optimization of the nanoplasmonic architectures is key to influencing the LSPR properties that fundamentally affect optical absorption, scattering, and near-field enhancements in device applications. To evaluate these design parameters, we employ FDTD simulations—a powerful computational tool that solves Maxwell’s equations iteratively to model the electromagnetic interactions within complex nanostructures [24].

The basic building block for simulation is a metallic nanoparticle, nanodisk, or nanosemisphere in a periodic array on top of a thin-film device. The multi-coefficient model (MCM) is used to model the material dispersion of metal and Si. Unlike conventional models—such as the Drude or Lorentz models—that often fall short in capturing inter-band transitions and complex spectral features, the MCM uses a series of coefficients to account for both free-electron and bound-electron contributions. This results in realistic dielectric functions, ensuring that the simulations reliably predict the LSPR peak positions, absorption enhancements, and scattering behavior. The dielectric functions of Ag and Al are based on the measured data from Palik [25]. The optical properties data for nc-Si:H input into the software were from measurements performed on plasma-enhanced chemical vapor-deposited (PECVD) nc-Si:H films.

Due to the rapid spatial variations of the electromagnetic fields near metal–dielectric interfaces, a non-uniform grid with a high resolution (down to a few nm) in critical regions was implemented. This fine mesh ensures that the intense near-field enhancements associated with LSPRs are accurately captured, while coarser grids can be applied in regions where field variations are less pronounced [24].

Boundary conditions are implemented to minimize artificial reflections and to mimic an infinite periodic structure. We use perfectly matched layers (PMLs) at the top and bottom boundaries to effectively absorb outgoing waves, while periodic boundary conditions (PBCs) are applied on the lateral boundaries to simulate an infinite array of nanoparticles. Where applicable, symmetric or anti-symmetric boundary conditions further reduce the simulation domain, thus optimizing computational efficiency without compromising accuracy [24].

**Figure 1:**
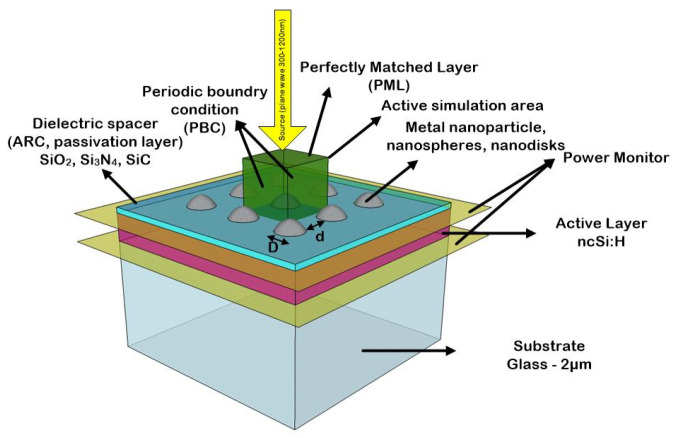
Schematic of proposed simulation building blocks. A periodic array of the metal nanosphere is used to evaluate absorption enhancement within the nc-Si:H layer. The periodic boundary condition and perfectly matched layer are used as the boundary conditions.

To model the illumination source, we used the AM1.5 spectrum, an incident plane wave with a wavelength range of 300 nm to 1100 nm that is typically used for solar cell characterization [26]. Two power monitors are used at the top and bottom of the absorbing layer for the absorption calculation (Figure 1).

The fraction of incident light absorbed in the nc-Si:H layer is calculated as follows:

(1) $$P_{abs\left(x,\lambda \right)}=\nabla .\left(\frac{1}{2}real\left(E\left(x,\omega \right)\times H\left(x,\omega \right)\right)\right)=\frac{1}{2}\omega {\left|E\left(x,\omega \right)\right|}^{2}Im\left(\varepsilon_{nc-Si:H}\left(\omega \right)\right)$$

where ω is the frequency of incident light, |E|^2 is the total power inside the layer, and Im(ε) is the imaginary part of permittivity. The total integrated absorption, ABST, within the spectral range of 300–1200 nm can be defined as

(2) $${ABS}_{T}=\int_{\lambda =300}^{\lambda =1100}P_{abs}\left(\lambda \right)I_{AM1.5}\left(\lambda \right)d\lambda$$

where I_AM1.5 (λ) is the AM1.5 solar spectrum obtained from the Renewable Resource Data Center [25]. To evaluate performance enhancement in design parameterization, integrated optical quantum efficiency (IQE) is used. This metric represents the overall photon-to-electron conversion efficiency of the device across the solar spectrum. It is obtained by integrating the spectral quantum efficiency, QE(λ), weighted by the incident AM1.5 solar spectrum, as defined in Equation (4). IQE is defined as,

(3) $$\begin{array}{c} IQE=\frac{Number\;of\;photons\;absorbed\;within\;nanocrustalline\;silicon\;thin\;film\left(300-1100\right)}{Total\;number\;of\;photons\;in\;sunlight\;\left(300-1100\right)}=\\ \frac{\int_{\lambda =300}^{\lambda =1100}\frac{\lambda }{hc}QE\left(\lambda \right)I_{AM1.5}\left(\lambda \right)d\lambda }{\int_{\lambda =300}^{\lambda =1100}\frac{\lambda }{hc}I_{AM1.5}\left(\lambda \right)d\lambda }\;\;\; \end{array}$$

where h is Plank’s constant, c is the speed of light in the free space, and QE(λ) is optical quantum efficiency calculated by

(4) $$QE\left(\lambda \right)=\frac{P_{abs}\left(\lambda \right)}{P_{sun}\left(\lambda \right)}$$

where Psun (λ) is the power of incident light. Therefore, the absorption enhancement ratio, η (λ), and the IQE enhancement ratio, EIQE, are defined to evaluate the efficiency of the metallic nanostructure

(5) $$\eta \left(\lambda \right)=\frac{{QE}_{plasmonic}\left(\lambda \right)}{{QE}_{bare}\left(\lambda \right)}$$

(6) $$E_{IQE}=\frac{{IQE}_{plasmonic}}{{IQE}_{bare}}$$

## 3. Results

**Figure 2:**
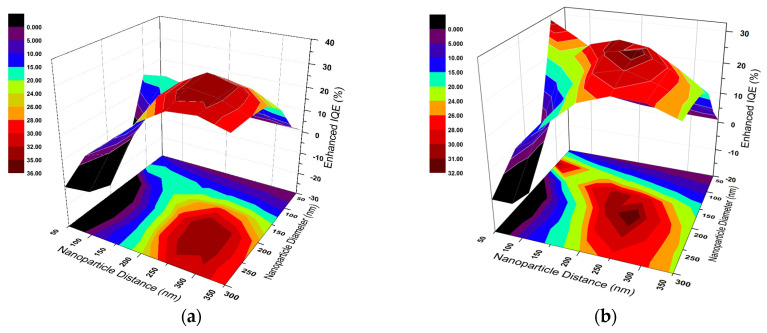
Enhanced integrated quantum efficiency (EIQE) of (**a**) silver, and (**b**) aluminum plasmonic nanoparticles on top of the proposed nc-Si:H film as a function of particle diameter (D) and particle spacing (d). The color scale corresponds to the EIQE, as defined in Equation (6), which is the ratio of integrated quantum efficiency with plasmonic enhancement to that of the reference (bare) nc-Si:H structure.

In order to assess the effect of nanoparticles, the variation in enhanced IQE (EIQE) is evaluated as a function of particle spacing (d) and particle diameter (D). The color maps shown in Figure 2 illustrate the enhanced integrated quantum efficiency (EIQE), defined in Equation (6), as the ratio between the IQE of the nanoplasmonic enhanced structure and that of the reference structure without nanoparticles. This ratio-based integrated metric enables a clear visualization of the relative performance improvements across varying nanoparticle diameters and spacings. Figure 2a,b present the IQE changes in a 300 nm-thick nc-Si:H film as a function of nanoparticle spacing (d) and diameter (D) for Ag and Al nanoparticles, respectively.

A peak enhancement of IQE is clearly seen at optimum d and D for each material. It may be noted that for Ag, the maximum IQE, which equals maximum optical absorption, is obtained for nanoparticle arrays with D = 200 nm and d = 250 nm with IQE = 0.4285. When compared to the IQE of 0.3171 for bare nc-Si:H (i.e., no Ag nanoparticles), it indicates a 35.15% increase in absorption. Further analysis shows that enhancements are low for small (D < 100 nm) and large (D > 300 nm) nanoparticles. This is due to light absorption by small nanoparticles and less effective forward scattering for large nanoparticles caused by high-order plasmon excitation, respectively [27]. Data for Al nanoparticles show that the maximum enhancement in absorption is by 31.91% for D = 200 nm and d = 250 nm with IQE = 0.4183.

**Figure 3:**
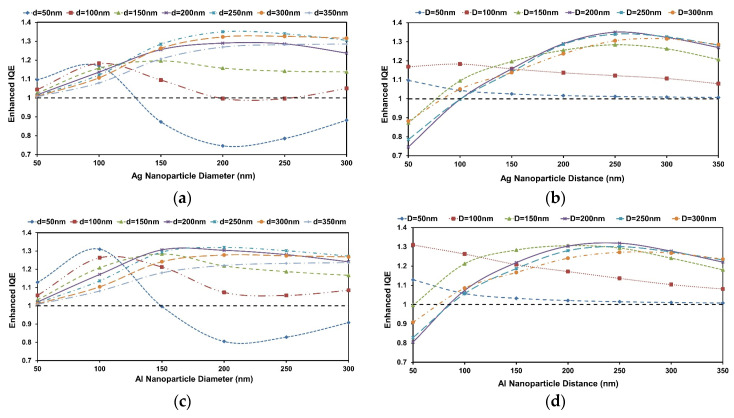
Enhanced integrated quantum efficiency ratio changes in Al/Ag nanoparticle arrays on 300 nm nc-Si:H/10 nmSiO_2_ structure. (**a**,**c**) based on different inter-particle distances for 200 nm nanoparticle; (**b**,**d**) based on different nanoparticle diameters for 250 nm inter-particle distance. The black dashed line is the reference structure (without nanoplasmonic array).

Figure 3a–d show the EIQE ratio variation for Ag and Al nanoparticle arrays. The dependence on D and d exhibits characteristic trends for both cases, showing the peak absorption maxima are reached for different D values and the positional dependence of the maxima also on d. The inter-particle distance at which the maximum absorption occurs for a given D shifts to a higher d value when D is increased. Interestingly, both metals almost show their maximum performance at the same nanoparticle array scheme of d = 250 nm and D = 200 nm. However, Al yields a higher EIQE at small particle diameter.

This optimal configuration results from a balance between efficient forward scattering and minimized parasitic absorption. Notably, for small D values (≤100 nm), the EIQE is lower due to dominant absorption losses within the particles, while at large D values (≥300 nm), higher-order multipole resonances and phase retardation effects reduce the radiative efficiency. As d increases beyond optimal spacing, inter-particle coupling weakens, diminishing waveguide mode excitation and light trapping. These trends indicate the critical role of both near-field coupling and resonance tuning in achieving broadband performance enhancement.

In order to explain the observed results, the mechanistic view of surface plasmon resonance in metal nanoparticles is key [28,29,30,31,32]. For small metallic nanoparticles, scattering processes are usually negligible, and the particle absorbs energy through three major mechanisms: (i) collective excitations of the “free” electrons, which give rise to surface modes or surface plasmon resonance determined by the particle shape, size, environment, and material composition; (ii) electronic transitions of bound electrons from occupied to empty bulk bands of different indexes, also called inter-band transitions; and (iii) surface dispersion or scattering of the “free” electrons, when their mean free path is comparable to the dimension of the nanoparticles. These mechanisms are the dominant reasons for decreasing enhancement in quantum yield by increasing metal nanoparticle size. For larger D values, the assumption of a uniform external field across the entire particle becomes invalid. Dynamic depolarization arises from non-uniform phase distributions within the particle, while retardation effects occur when the particle’s dimensions become comparable to the wavelength of light. Both effects reduce the effective dipolar response, thereby diminishing radiative efficiency and forward scattering, and lead to an increased likelihood of parasitic absorption [33].

It is notable that Al consistently exhibits higher EIQE compared to Ag under similar array conditions. This behavior is attributed to Al’s plasmonic resonance occurring in the UV region, which leads to minimal parasitic absorption in the visible range—where the solar spectrum is most intense. In contrast, Ag exhibits stronger parasitic absorption due to its LSPR aligning with visible wavelengths, thereby reducing net absorption in the nc-Si:H layer. Additionally, Al provides more broadband scattering and less pronounced damping in the operational spectral range, further improving integrated optical performance [34].

**Figure 4:**
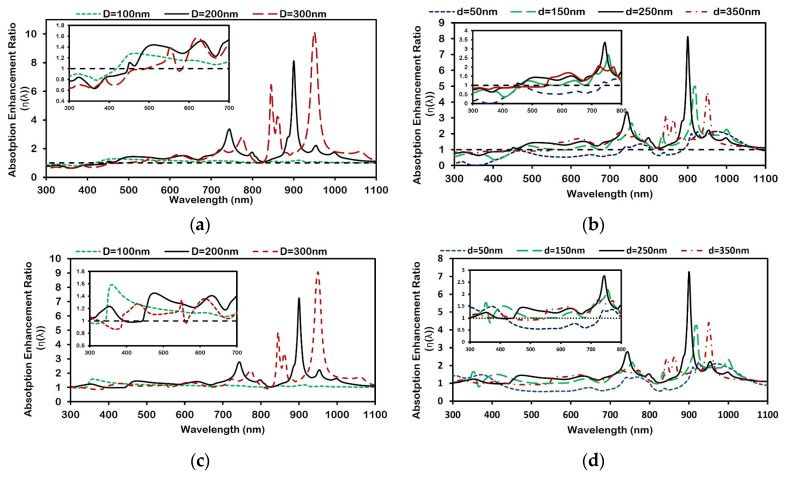
Absorption enhancement ratio for Ag (**a**,**b**) nanoparticles and Al (**c**,**d**) nanoparticles. (**a**,**c**) Nanoparticle arrays with an inter-particle distance of 250 nm and different diameters; (**b**,**c**) nanoparticle array with a 200 nm diameter and different inter-particle distances. The inset shows the absorption enhancement at a short wavelength (300–800 nm). The black dashed line at a ratio value of 1 represents the reference nc-Si:H structure without the nanoplasmonic array.

The variation in the absorption enhancement ratio η(λ) as a function of wavelength is shown in Figure 4a–d for different schemes of Ag and Al nanoparticle arrays. The peaks in Figure 4 represent wavelengths where absorption enhancement is maximized due to resonant interactions, primarily LSPR and waveguide mode excitation. As nanoparticle diameter increases (Figure 4a,c for Ag and Al respectively), the dominant resonance red-shifts (moves to a higher wavelength) due to stronger retardation and multipolar effects. Conversely, increasing inter-particle distance (Figure 4b,d for Ag and Al respectively) reduces plasmonic coupling, resulting in a blue shift (moving to a lower wavelength) in the enhancement peaks. Multiple peaks observed at larger diameters are attributed to higher-order plasmon modes and increased scattering complexity. It is clear from Figure 4a,c that as particle diameter increases from 100 nm to 300 nm, with a fixed d of 250 nm, the number of absorption enhancement peaks increases. This contributes to the increase of local surface plasmon resonance (LSPR) modes in coupled metallic nanoparticle arrays [1,34]. The presence of these multipole modes in coupled nanoparticles can be attributed to a non-uniform incident field driving the LSPR. These excite LSPRs, inducing polarization currents in individual particles that radiate into the underlying structure. If the thickness of a spacer layer (e.g., SiO_2_) is small, the radiating nanoparticles transfer a significant portion of their energy into the waveguide modes of the absorber layer (nc-Si:H). The particle–waveguide coupling enhances absorption in the thin Si layer because the coupled light is “trapped” by the guided modes, in contrast with light trapping in crystalline Si solar cells [35].

Also, the wavelength at which the absorption enhancement ratio becomes <1 (i.e., the absence of enhancement) shifts towards a higher wavelength for increasing nanoparticle size—a red shift in resonance. As D is increased for a fixed d, two factors must be considered: first, the incident external driving field about the particle will become less uniform, and second, the inter-particle coupling becomes stronger because of the reduced gap between the surfaces of the particles. Both factors increase dynamic depolarization, which ultimately gives rise to the multipole LSPR mode.

Figure 4b,d describe the spectral variation in the absorption enhancement ratio for different inter-particle array distances between 150–350 nm for a fixed D of 200 nm. It is clear that as the inter-particle distance increases from 150 nm to 350 nm, a blue shift is observed in the wavelength at which maximum absorption enhancement occurs. Hence, as the arranged nanoparticles are brought closer, keeping their size, shape, and other parameters constant, the change in overall absorption behavior within nc-Si:H corresponds to the change in the inter-particle coupling between them.

In arrays of metallic nanoparticles, the strength of plasmon coupling is strongly dependent on the inter-particle separation (d). At relatively large d, the interaction mimics that between two classical dipoles, with a coupling potential that decays as 1/d^3^ [36]. Under these conditions, radiative damping of the induced dipole predominates, yielding a single, well-defined dipolar resonance. Conversely, as the particles are brought closer together, their near-field interactions intensify. The electromagnetic fields of adjacent particles begin to overlap significantly, leading to a superposition of the external driving field with strong near-field contributions. This superposition confines the net electromagnetic field in the gaps between particles, resulting in a spatially non-uniform excitation across each nanoparticle. Consequently, dynamic depolarization effects become more pronounced, facilitating plasmon hybridization. Here, the original dipolar modes split into multiple hybridized modes—a process that manifests as a red shift in the localized surface plasmon resonance (LSPR) due to enhanced dephasing and the mixing of higher-order multipole oscillations.

This transition from a single dipolar resonance at large separations to a red-shifted, hybridized plasmonic response at small separations highlights the interplay between radiative damping, dynamic depolarization, and near-field coupling in governing the optical properties of nanoparticle arrays [36].

The red or blue shift in arrays of metal nanoparticles in comparison to single-particle spectra has already been reported when polarized light is used to illuminate the structure depending upon the angle of illumination and polarization [37,38]. Here, it is shown that by illuminating the structure with unpolarized light, the effect of increasing inter-particle coupling between nanoparticles shifts the peak position in the absorption enhancement curve. As explained before, this behavior is attributed to the decaying field distribution between consecutive particles as near-field inter-particle coupling decreases with increasing d.

**Figure 5:**
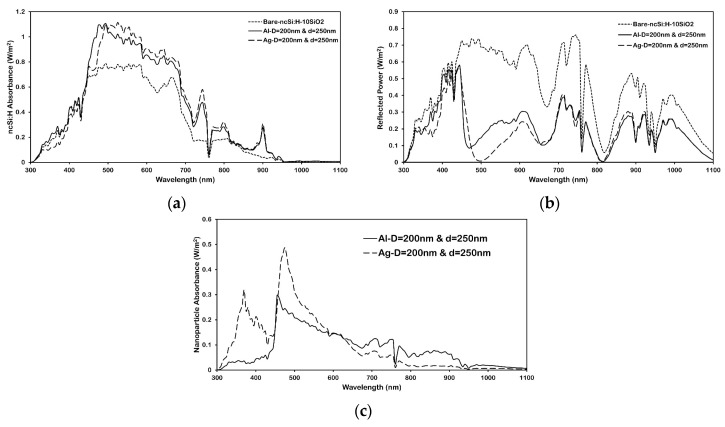
Spectral characteristics of (**a**) nc-Si:H absorbance (**b**), reflection (**c**), and nanoparticle absorbance of the 200 nm diameter Al and Ag nanoparticles with a particle distance of 250 nm placed on the front surface of a 300 nm-nc-Si:H/10 nm-SiO_2_ structure.

**Figure 6:**
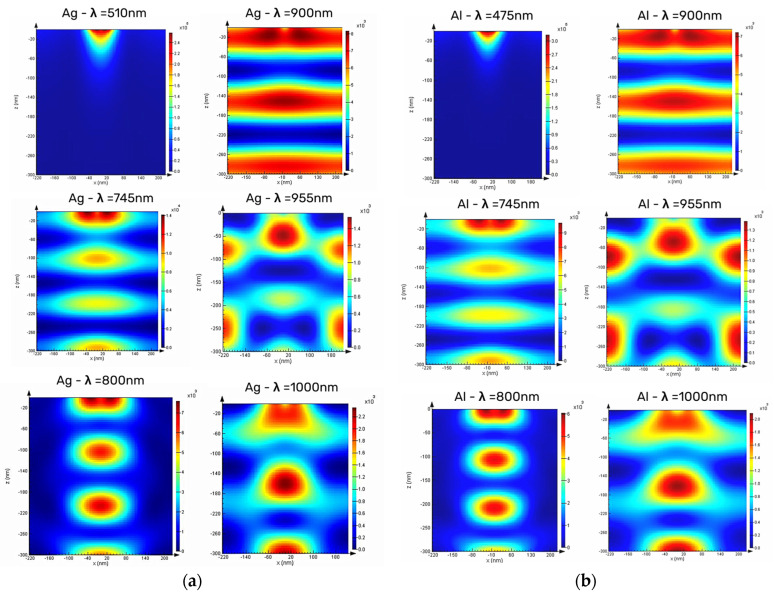
The optical absorption profile per unit volume for the 300 nm-nc-Si:H active layer in the presence of (**a**) Ag and (**b**) Al nanoparticle arrays, with a 200 nm diameter and 250 nm inter-particle distance at different wavelengths.

Figure 5a–c show the total power absorption in 300 nm nc-Si:H film, the total reflection power, and total absorption in metallic nanoparticles, respectively. It is apparent from Figure 5a that a broadband absorption enhancement in nc-Si:H occurs for both Al and Ag, a change attributed to the dramatically reduced light reflection shown in Figure 6b. However, absorption in the nc-Si:H film in the presence of Ag nanoparticles decreases in the short wavelength as compared to Al. This phenomenon is due to the strong parasitic light absorption near the surface plasmon resonance wavelength, as shown in Figure 5c.

Al nanoparticles have smaller parasitic absorption in the shorter wavelength region since the surface plasmon resonance of Al nanoparticles lies in the ultraviolet region, where the intensity of AM1.5 solar irradiance is small. Figure 5b compares broadband reflection spectra of Ag and Al nanoparticle arrays with bare nc-Si:H. The reflection for base nc-Si:H film (blur dot line) shows strong oscillation that becomes broader with increasing wavelength. These result from the Fabry–Pérot effect in the different layers, which occurs when the structure behaves like a waveguide with the active layer (nc-Si:H), with the refractive index of ≈ 3.9 being located between the spacer (n ≈ 1.4) and glass substrate (n ≈ 1.5). The particle array gives a broadband reduction in reflectivity. The Fabry–Pérot peaks in the reflection spectrum are reduced because the particles preferentially scatter the reflected light back into substrates.

Furthermore, the occurrence of sharp dips at some wavelengths such as 745 nm and 900 nm, which is in parallel with sharp increases in absorption spectrum, reveals that the particle array generates enough in-plane momentum to couple light into waveguide modes of the active layer. To prove that the sharp dips in reflection are due to mode coupling, the fraction of incoming power that is absorbed in the 300 nm nc-Si:H active layer is calculated using Equation (1). In order to further explore the broadband absorption enhancement, the profile of optical absorption per unit volume (µm^3^) in the active layer at different wavelengths for Ag and Al nanoparticles on top of the 300 nm-nc-Si:H/10 nm-SiO_2_ structure are shown in Figure 6.

In Figure 6, the absorption profile at different wavelengths clearly reveals regions of strong local absorption with distinct spatial features, appearing as both circular and linear profiles. These patterns are indicative of complex nanoplasmonic interactions, particularly the coupling of light to the nanoparticle array and the formation of absorptive “hot spots”.

The electromagnetic fields scattered by adjacent nanoparticles can interfere constructively or destructively, known as inter-particle coupling and interference. At optimal inter-particle distances, constructive interference enhances the coupling of light into waveguide modes, whereas too-close spacing can lead to destructive interference or overcoupling, which confines the light between particles rather than directing it efficiently into the absorber [39,40]. The occurrence of forward light scattering towards the active layer at wavelengths of 510 nm and 475 nm are clear for both Ag and Al, respectively. Also, for both Ag and Al, the nanoparticles scatter incident light into the substrate, resulting in a local intensity enhancement and thereby a local absorption enhancement at the location of particles (x = 0).

Additionally, the light coupling to the waveguide mode—referred to as waveguide mode excitation (WME)—in the nc-Si:H layer, which propagates through the waveguide in the horizontal direction, is also evident. This, in turn, causes an oscillating intensity profile as a function of x, with a wavelength that depends on the inter-particle distance. This can be attributed to the evanescent tail of the waveguide mode interacting with the particle array, thereby experiencing a periodically changing effective refractive index. This gives rise to the formation of a Bloch mode, as illustrated in Figure 6a for a wavelength of 955 nm. Both Figure 6a,b also suggest that light couples to a waveguide mode that propagates in the in-plane direction. At this point, it may be concluded that investigating the waveguide mode in the active region—specifically for ultrathin layers—can be a very helpful tool for the design and performance prediction of device applications in photovoltaic, high-sensitivity sensing, integrated photonics, and light-emitting technologies.

An important practical consideration when utilizing aluminum nanoparticles for plasmonic applications is their susceptibility to rapid oxidation in ambient environments, leading to the formation of a native Al_2_O_3_ oxide shell. This oxide layer can shift the LSPR peak position, broaden the resonance linewidth, and diminish the near-field intensity, potentially affecting the optical performance of plasmonic-enhanced thin-film devices. Recent studies, such as those by Pujari et al. [41] and Ziashahabi et al. [42], have reported similar observations, noting significant alterations in resonance behavior due to aluminum oxidation. To mitigate these challenges, strategies such as alloying aluminum nanoparticles with other earth-abundant metals, employing ultra-thin protective coatings (e.g., SiO_2_, Al_2_O_3_ via atomic layer deposition), or immediate encapsulation techniques can be adopted. Such approaches help maintain desired plasmonic properties and enhance long-term device stability.

### 3.1. Effect of Spacer Dielectric Materials and Its Thickness

**Figure 7:**
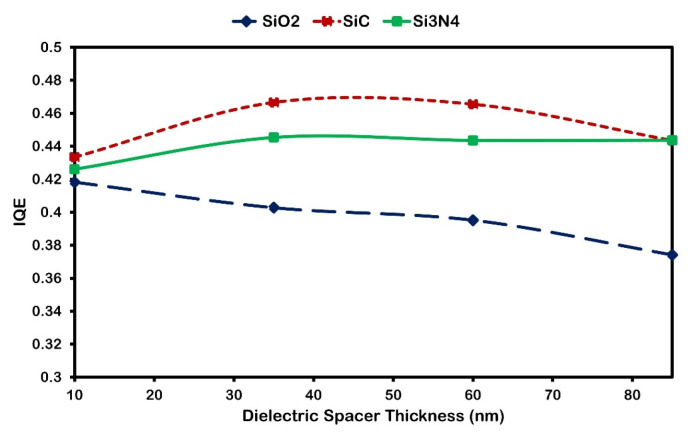
Integrated quantum efficiency of 300 nm nc-Si:H solar cell Al nanoparticles with a 200 nm diameter and 250 nm inter-particle array distance on the top layer and different dielectric spacer thicknesses and materials.

The dielectric properties of the surrounding medium, including substrate and spacer layers, significantly influence modulating LSPRs and their coupling to waveguide modes in the nc-Si:H layer. This sensitivity to the dielectric environment is key for tuning the optical response and optimizing device performance [39]. Here, we systematically examine the influence of spacer materials—SiC, Si_3_N_4_, and SiO_2_—and their thicknesses (10–85 nm) on the integrated quantum efficiency (IQE) and absorption enhancement ratio η(λ). As shown in Figure 7, SiC and Si_3_N_4_ generally exhibit higher IQE compared to SiO_2_. This outcome stems from their relatively low refractive indices and effective anti-reflection properties, which help maintain strong near-field coupling over a broader range of thicknesses.

By contrast, when the thickness of the SiO_2_ spacer increases, the nanoparticles become more distanced from the nc-Si:H layer, weakening the near-field coupling and thereby reducing IQE. However, this effect is less pronounced for SiC and Si_3_N_4_ at moderate thicknesses (30–40 nm), where the beneficial interplay between anti-reflection and plasmonic coupling is still preserved.

**Figure 8:**
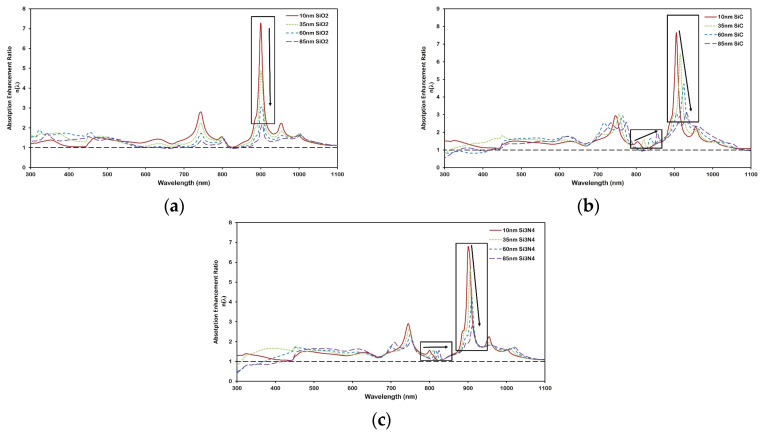
Absorption enhancement ratio η(λ) for Al nanoparticles (D = 200 nm, d = 250 nm) on 300 nm nc-Si:H with varying thicknesses of (**a**) SiO_2_, (**b**) Si_3_N_4_, and (**c**) SiC spacer layers. As spacer thickness increases, absorption enhancement decreases due to weaker near-field coupling. The black dashed line at a ratio value of 1 represents the reference nc-Si:H structure without the nanoplasmonic array.

**Figure 9:**
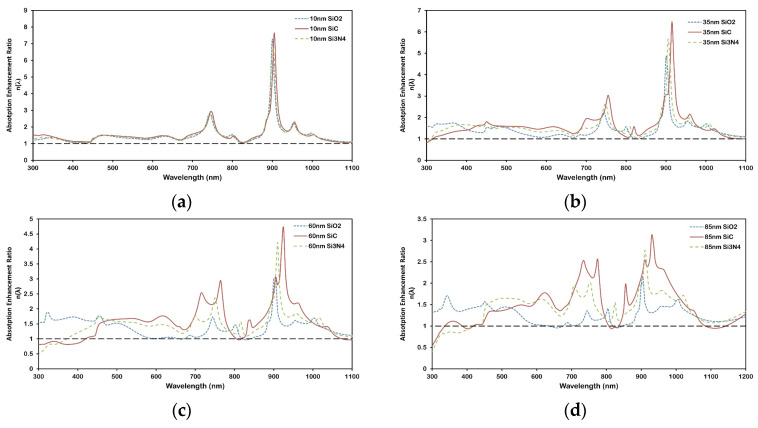
Effect of different spacer (SiO_2_, SiC, and Si_3_N_4_) on spectral shift in absorption enhancement ratio η(λ) for Al nanoparticles on 300 nm nc-Si:H for (**a**) 10 nm, (**b**) 35 nm, (**c**) 60 nm, and (**d**) 85 nm spacers thickness. Red shift in peaks is observed for higher-index materials, indicating modified waveguide–plasmon coupling. The black dashed line at a ratio value of 1 represents the reference nc-Si:H structure without the nanoplasmonic array.

Figure 8 and Figure 9 illustrate how the spacer material and its thickness impact the spectral characteristics of plasmonic enhancement in the nc-Si:H layer. Both figures present the absorption enhancement ratio η(λ), with Figure 8 showing the overall trend with thickness for each spacer material, and Figure 9 highlighting the spectral shifts of enhancement peaks. It is observed that as the spacer thickness increases, the magnitude of enhancement decreases for all materials due to reduced near-field coupling between the nanoparticles and the absorber layer.

Importantly, Figure 9 reveals a red shift in the enhancement peaks for Si_3_N_4_ and SiC, but not for SiO_2_. This shift highlights the sensitivity of LSPR and waveguide mode excitation to changes in the local dielectric environment [40,43]. The magnitude of this shift differs among SiC, Si_3_N_4_, and SiO_2_, underscoring the importance of choosing spacer materials and thicknesses that align with the target operating wavelength range. Higher-index materials like Si_3_N_4_ and SiC increase the modal confinement and shift the coupling condition between scattered light and waveguide modes to longer wavelengths. In contrast, SiO_2_, with its lower refractive index, causes minimal perturbation to the optical field distribution and exhibits no significant spectral shift. This highlights the critical role of the dielectric environment in tuning the hybrid plasmonic-photonic resonances for broadband device optimization.

From a broader nanoplasmonic perspective, this tunability of resonance position and intensity enables fine control over light scattering and near-field enhancement, which can be leveraged in ultrathin photovoltaic devices, plasmonic sensors, and integrated photonic systems. Also, by balancing spacer thickness and refractive index, one can optimize forward scattering into the active layer while minimizing parasitic losses.

### 3.2. Effect of nc-Si:H Active Layer Thickness

To investigate the effect of active layer thickness, absorption enhancement assessment is performed when nc-Si:H films of different thicknesses—100 nm–300 nm—are integrated with an Al nanoparticle array (D = 200 nm, d = 250 nm) and a 10 nm SiO_2_ spacer.

**Figure 10:**
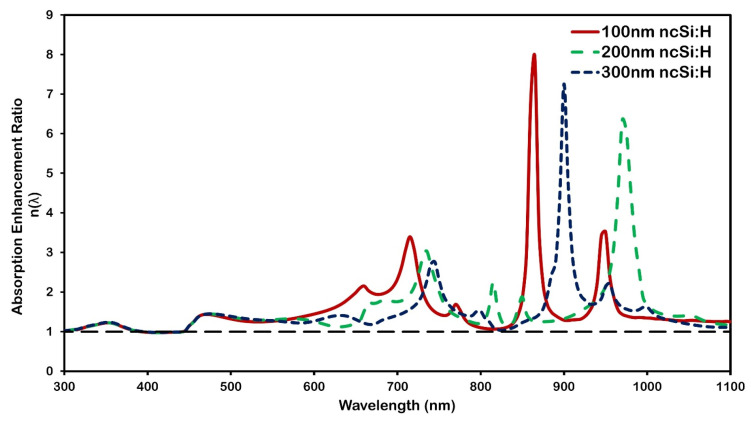
Absorption enhancement ratio variation with wavelengths for Al nanoparticle arrays, which have a 200 nm diameter and 250 nm inter-particle distance on top of the solar cell with 10 nm SiO_2_ as the dielectric spacer and three distinct active nc-Si:H thicknesses. The black dashed line at a ratio value of 1 represents the reference nc-Si:H structure without the nanoplasmonic array.

As shown in Figure 10, which depicts the area beneath the absorption spectrum curve, total absorption generally reduces by decreasing the active layer thickness. However, it reveals that absorption enhancement happens more intensely within the thinner nc-Si:H layer. This is related to the increasing density waveguide mode for a specific wavelengths, as thickness decreases. Additionally, absorption enhancement at a longer wavelength is more effective for a thinner absorber, as shown in Figure 10.

In general, by lowering active layer thickness, the overall absorption decreases owing to lower material volume. Figure 10 shows that while total absorption generally decreases with decreasing nc-Si:H thickness in the 300–800 nm range, the behavior in the longer wavelength region (800–1100 nm) is reversed. Specifically, the absorption enhancement ratio η(λ) becomes higher for thinner absorbers (100 nm), indicating stronger plasmon-induced trapping at infrared wavelengths. This counterbalance arises because ultrathin layers support a denser distribution of waveguide modes, which more effectively couple scattered light into the absorber at specific wavelengths. Additionally, thinner nc-Si:H films exhibit stronger absorption enhancement in the longer-wavelength region due to improved modal overlap with the guided modes. In contrast, thicker layers (e.g., 300 nm) exhibit high intrinsic absorption in the visible range and are less sensitive to plasmonic effects, resulting in a lower η(λ). The spectral shifts observed across different thicknesses reflect changes in waveguide dispersion and modal overlap with the scattered near fields. Quantitatively, the nanoparticle array boosts the absorption by 44.5% for a 100 nm nc-Si:H layer, compared to 35.9% for 200 nm and 31.9% for 300 nm layers. These findings highlight that while thicker layers inherently absorb more light, the plasmon-induced enhancement mechanisms are most effective in ultrathin configurations, offering significant advantages for applications in lightweight photovoltaic and other optoelectronic devices [43].

### 3.3. Effect of Metallic Object Shape

The geometry of metallic nanostructures plays a critical role in dictating their plasmonic behavior and, consequently, the optical performance of the device. We compared effect of three geometries of Al —spherical nanoparticles, nanosemispheres, and nanodisks—on the absorption enhancement of a 300 nm nc-Si:H layer with10 nm SiC spacer.

**Figure 11:**
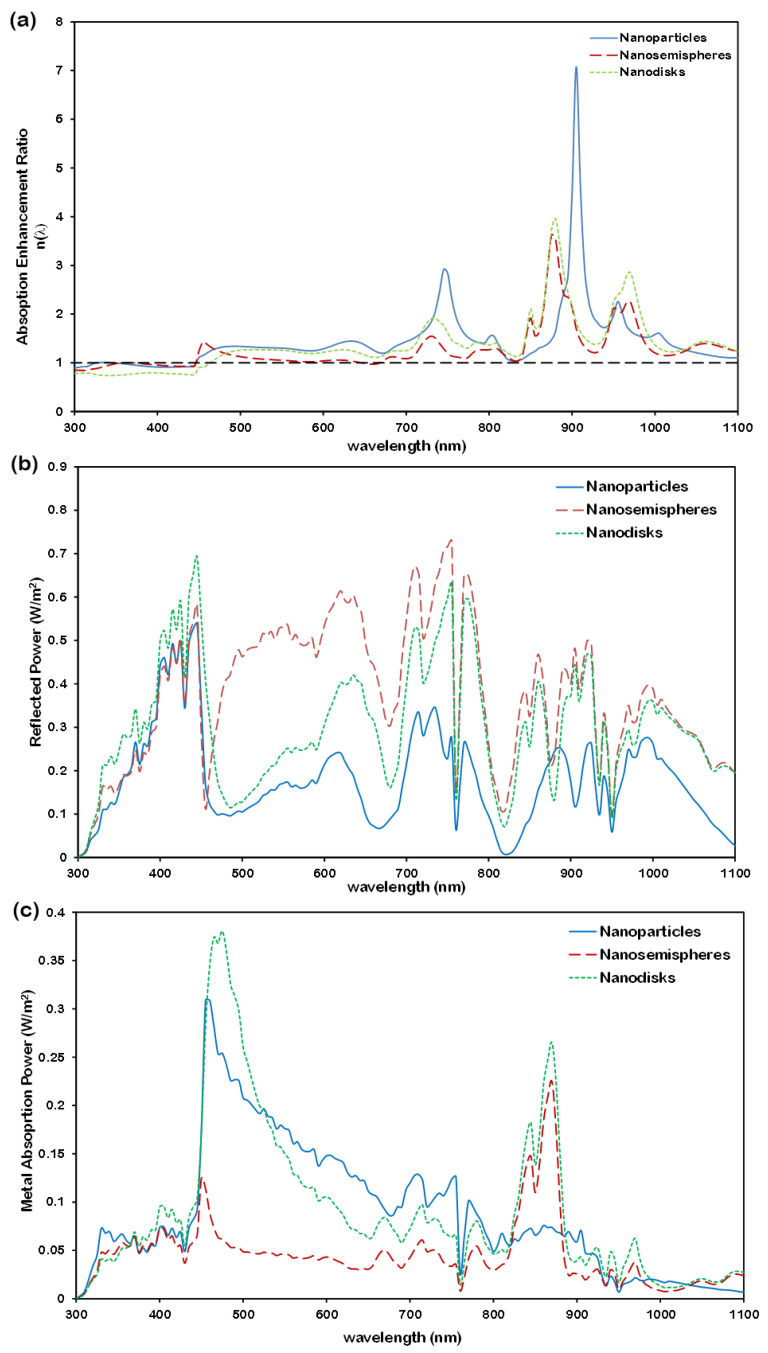
Effect of nanoparticle shape array on (**a**) absorption ratio variation with wavelength (the black dashed line at a ratio value of 1 represents the reference nc-Si:H structure without the nanoplasmonic array), (**b**) reflection ratio spectrum, and (**c**) absorption ratio in metallic array. Array parameters are 200 nm-diameter Al nanoparticles with a 250 nm inter-particle distance (nanoparticles), 200 nm-diameter Al nanosemispheres with a 250 nm inter-particle distance (nanosemispheres), and 200 nm-diameter Al nanodisks with 100 nm in length and 250 nm inter-disk distance.

Figure 11a shows that spherical nanoparticles yield the highest absorption enhancement in the nc-Si:H layer, particularly with strong peaks at 750 nm and 905 nm, and an overall broadband improvement over the 450–700 nm range. This performance is attributed to the superior ability of spherical geometry to induce strong forward scattering, thereby efficiently directing light into the absorber.

In contrast, nanosemispheres tend to reflect a larger fraction of incident light, as evidenced by the higher total reflection ratio in Figure 11b. This increased reflection limits the net power coupled into the active layer, despite their relatively low parasitic absorption. Nanodisks, while performing better than nanosemispheres, do not match the forward scattering efficiency of spherical nanoparticles. Figure 11c illustrates that the power absorbed within the metallic array is higher for spherical nanoparticles, reinforcing their advantage in enhancing overall device absorption.

The observed differences can be attributed to the impact of shape on the localized surface plasmon resonances (LSPRs). Spherical nanoparticles support well-defined dipolar modes with minimal geometric distortion, leading to effective radiative coupling and strong near-field enhancement. In contrast, the asymmetry inherent in nanosemispheres and the anisotropic nature of nanodisks can give rise to complex multipolar excitations that either spread the electromagnetic energy or redirect it away from the active layer.

These insights suggest that optimizing the metallic nanoparticle shape—potentially through surface modifications to minimize reflection in non-ideal geometries—could further improve light coupling efficiency. Beyond photovoltaics, such control over plasmonic resonances and scattering directionality is valuable in photodetector design, plasmon-enhanced light-emitting devices, and sensing applications, where tailored electromagnetic field distributions are essential for performance enhancement.

This study provides a comprehensive theoretical framework for optimizing nanoplasmonic metallic nanoparticle arrays to significantly enhance optical absorption in nc-Si:H devices. The observed enhancement trends—including resonance tuning, waveguide mode coupling, and spacer-induced spectral shifts—are consistent with experimental results reported in previous studies involving similar plasmonic nanostructures [3,9,11]. Such alignment highlights the practical relevance and validity of the parametric approach established in this work.

## 4. Conclusions

This comprehensive investigation and design parameterization of nanoplasmonic architectures in ultrathin nc-Si:H films not only establishes a robust framework for enhancing photovoltaic performance but also lays the groundwork for a broad spectrum of optoelectronic applications. By systematically optimizing key design parameters—including metallic material selection, nanoparticle geometry, inter-particle spacing, and dielectric spacer properties—we demonstrate that nanoplasmonic arrays can significantly boost light absorption through multiple synergistic mechanisms.

Our numerical simulations, conducted via advanced FDTD methods, reveal that appropriately engineered metallic nanoparticle arrays enhance absorption efficiency by 25–30% in ultrathin silicon films (100–200 nm). This enhancement arises from (i) the effective scattering of incident light by subwavelength metallic particles, which increases the optical path length through light-folding, (ii) the excitation of propagating waveguide modes within the thin absorber, and (iii) the generation of intense near-fields via localized surface plasmon resonances at the metal/semiconductor interface. Notably, our comparative analysis indicates that Al outperforms Ag in broad-spectrum light trapping, particularly at wavelengths aligned with the nc-Si:H band gap, while spacer materials such as SiC yield superior absorption enhancement relative to SiO_2_ and Si_3_N_4_.

Building upon these theoretical insights, future research might concentrate on the experimental implementation of the optimum designs investigated in this study. A key activity and challenge for upcoming work will be the development of suitable fabrication techniques for the proposed nanoplasmonic architecture, such as advanced lithography methods combined with thin-film deposition processes. This integrated theoretical-experimental strategy will further enhance the practical relevance, credibility, and applicability of the presented nanoplasmonic optimization approach.

The design principles and plasmonic phenomena underpinning enhanced absorption can be exploited to improve the performance of advanced photovoltaic, photodetection, light-emitting, and sensing platforms. For instance, the ability to tailor local field enhancements and control light–matter interactions is critical for developing high-sensitivity biosensors and integrated photonic circuits. In summary, this work underlines the critical role of precise nanoplasmonic parameterization in optimizing the architectures in advancing next-generation optoelectronic devices.

## Data Availability

The authors confirm that the data supporting the fundings of this study are available within the article.
